# Monozygotic twinning following embryo biopsy at the blastocyst stage

**DOI:** 10.5935/1518-0557.20200069

**Published:** 2021

**Authors:** Rafael Sellers, Juan Carlos Castillo, Jorge Ten, Adoración Rodríguez, José A. Ortiz, Francisco Sellers, Joaquín Llácer, Rafael Bernabeu

**Affiliations:** 1 Reproductive Medicine. Instituto Bernabeu, Alicante, Spain

**Keywords:** monozygotic twinning, embryo biopsy, blastocyst, preimplantation genetic testing

## Abstract

**Objective:**

Monozygotic twinning incidence following preimplantation genetic testing in embryos at cleavage-stage does not appear to increase; however, data regarding the possible impact of the blastocyst-stage preimplantation genetic testing is lacking. We compared the incidence of monozygotic twinning in preimplantation genetic testing cycles performed at the blastocyst-stage, *versus* cycles without PGT, following single embryo transfer.

**Methods:**

In this retrospective cohort study, we analyzed the incidence of twin pregnancies in patients undergoing intracytoplasmic sperm injection and blastocyst-preimplantation genetic testing (253 cycles), *versus* a period-matched control population of patients undergoing intracytoplasmic sperm injection and single embryo transfer without preimplantation genetic testing (606 cycles).

**Results:**

The overall monozygotic twinning rate was 14/859 (1.6%) per clinical pregnancy. The incidence of zygotic splitting following intracytoplasmic sperm injection and preimplantation genetic testing was 3.5% (95% Confidence interval 1.8%-6.6%) *versus* 0.8% (95% Confidence interval 0.3%-1.9%) following intracytoplasmic sperm injection without preimplantation sperm injection. After adjusting for potential confounders, preimplantation genetic testing cycles were associated with an increase in the incidence of monozygotic twinning when compared to cycles without embryo biopsy (Odd ratio 3.44, 95% Confidence interval 1.05-11.27, *p*=0.041).

**Conclusions:**

Our findings indicate that embryo biopsy for preimplantation genetic testing performed at the blastocyst stage is associated to an increase in the incidence of monozygotic twinning. Further validation in larger sample size studies is warranted. Patients undergoing preimplantation genetic testing must receive proper counselling about the potential risks of the technique.

## INTRODUCTION

Monozygotic twinning (MZT) (the splitting of a single fertilized oocyte into two or more fetuses) occurs in 0.42% of spontaneous pregnancies (Bulmer, 1970). Monozygotic twin pregnancies are associated with an increased risk of maternal and fetal complications, such as fetal growth restriction, preterm delivery and perinatal mortality (Derom *et al*., 1987; Malone, 2003). The exact origin of MZT in spontaneous pregnancies remains largely unknown and with the advent of assisted reproduction techniques (ART), several publications have raised concerns regarding the potential for an increased rate of MZT following ART (Abusheikha *et al*., 2000; Schachter *et al*., 2001). Recently, 1.5% monozygotic twin live birth rate following ART was reported (Mateizel *et al*., 2016), which appears concordant with the results from a large registered ART data analysis reporting a prevalence of 1.36% of multiple pregnancies with zygotic splitting even after single embryo transfer (Ikemoto *et al*., 2018). Additional publications have investigated different ART parameters and their putative involvement in embryo splitting and their potential association with MZT. This includes: maternal age (Ikemoto *et al*., 2018), ovulation induction (Derom *et al*., 1987), embryo culture conditions (Edwards *et al*., 1986), changes to the zona pellucida (ZP) from ICSI and/or assisted hatching (AH) (Hershlag *et al*., 1999), cryopreservation and prolonged embryo culture up to blastocyst stage (Nakasuji *et al*., 2014). Taken all together, the body of available evidence suggests an (almost uniform) association between blastocyst transfer and a potential risk of increased MZT; whereas frozen embryo transfer (FET) appears associated with a lower MZT rate. Other ART parameters appear equivocal and warrant future studies (Mateizel *et al*., 2016; Ikemoto *et al*., 2018).

Recently, following a revision in ART terminology (Zegers-Hochschild *et al*., 2017), the term “preimplantation genetic testing” (PGT) was introduced, to describe the test performed to analyze the DNA from oocytes (polar bodies) or embryos (cleavage stage or blastocyst) for HLA typing or for determining genetic abnormalities. Publications on the incidence of MZT after the use of PGT in ART cycles are scarce. At least theoretically, the incidence of MZT in PGT cycles must be potentially increased because of different mechanisms, including: intentional holes in the ZP and cell manipulation for biopsy. To the best of our knowledge, the only available publication on the topic reported that the incidence of MZT did not increase in PGT cycles compared with regular intracytoplasmic sperm injection with blastocyst transfer cycles (Verpoest *et al*., 2009), this publication included cleavage-stage embryo biopsies exclusively. More recently, cleavage-stage embryo biopsies have been largely replaced by trophectoderm (TE) biopsies. Blastocyst biopsy provides more cells and is performed at an embryonic stage more amenable to genetic analyses, and less sensitive to possible damage (Coll *et al*., 2018). We lack reports on the incidence of MZT after the use of ART in combination with PGT performed at the blastocyst stage.

The aim of this study was to assess the incidence of MZT pregnancies in a cohort of consecutive PGT treatment cycles performed at the blastocyst stage, followed by single blastocyst embryo transfer, in comparison with a period-matched cohort of consecutive ICSI cycles with single embryo transfer (SET) at the same center.

## MATERIAL AND METHODS

In this retrospective comparative cohort study, we analyzed the incidence of MZT pregnancies in a cohort of consecutive ICSI treatment cycles combined with PGT by TE biopsy and single blastocyst embryo transfer (study group) and compared the results with a control cohort of consecutive ICSI cycles and SET without embryo biopsy (control group). The study was conducted at a single private center for reproductive medicine and included data from January 2014 to December 2017.

### Study and control groups

ART cycles included own-eggs and donor-eggs cycles. Specific characteristics of the ovarian stimulation process were not included for analysis but there were no differences in laboratory conditions vis-à-vis procedures between control and study groups, except for the AH (on day-3 of embryo development) and TE biopsy procedure for PGT in the study group. Once collected, the oocytes were incubated in a culture medium (Global Total for Fertilization, LifeGlobal^®^) until denudation for subsequent ICSI (usually two hours later). In the study group, ICSI was performed in order to prevent contamination with residual sperm DNA (Liebaers *et al*., 1998). The details of the ICSI procedure have been described previously (Lledó *et al*., 2006). Fertilization was assessed 16-18 hours after ICSI and the embryos obtained remained in one-step culture medium (Global Total, LifeGlobal^®^). Further development was evaluated in the morning of day 3 when laser-AH (Lykos^®^, Hamilton Thorne^®^) was performed as previously described (Lledó *et al*., 2006). At the blastocyst stage, the embryos were classified according to morphologic characteristics by ASEBIR (2015). Laser-assisted biopsy and aspiration was applied consistently to remove five to eight TE cells.

Within the study group, depending on clinical decision, biopsied embryos were either selected for fresh transfer the day after biopsy (study-fresh group), vitrifying supernumerary euploid embryos, or for elective complete vitrification (study-frozen group) and deferred embryo transfer. Genetic analysis was performed using array-CGH (which provides results within 24 hours) in the fresh group, whereas Next Generation Sequencing (NGS) was used in the frozen group. Similarly, in the control group and depending upon clinical decision, embryos were selected either for fresh transfer (control-fresh group) plus vitrification of surplus embryos or for elective complete vitrification (control-frozen group) for deferred embryo transfer. A single embryo transfer was performed in all cases; cleavage or blastocyst stage transfer was allowed in the control group.

Endometrial preparation in frozen-thawed embryo transfer (FET) artificial cycles (exogenous estrogen administration plus timely addition of progesterone) as well as natural cycle endometrial preparation used as per clinical decision. Specific characteristics of the endometrial preparation process were not included in the analysis.

### MZT diagnosis

A clinical pregnancy was defined as one or more gestational sacs seen via transvaginal ultrasound scan at least 5 weeks after embryo transfer. MZT was identified at first ultrasound when the number of fetal heartbeats exceeded the number of gestational sacs, or when the number of sacs exceeded the number of embryos transferred.

### Statistical analysis

For the statistical analysis of patients and cycle characteristics, continuous variables were presented as number of cases, mean and typical deviation. A *p*=0.05 was considered statistically significant after performing a t-student test. Categorical variables were presented as number of cases, percentage and odds ratio (OR). Pearson's Chi-square test (univariate) and binary logistic regression (multivariate for confounding factors) were used to analyze association of specific ART features and MZT. A *p*-value was considered significant if <0.05. The statistical analysis was performed using the SPSS 20.0 software (SPSS, Chicago, IL, USA).

## RESULTS

Overall, we had 859 clinical pregnancies after SET during the study period. A total 606 cycles corresponded to ICSI and single embryo transfer without PGT and 253 cycles to patients undergoing ICSI and PGT (for various indications) with single blastocyst transfer. Of these, 14 resulted in twin pregnancies (global MZT rate 1.6% per clinical pregnancy). Globally, 93.3% of the cycles included embryo transfer at the blastocyst stage. Table 1 depicts the distribution of the baseline cycle characteristics within the MZT and singleton groups. Monozygotic splitting occurred significantly more frequently (*p*=0.004) in PGT cycles. Frozen embryo transfer cycles were found more frequently associated with MZT without reaching statistical significance (*p*=0.06).

**Table 1:** Age and specific cycle characteristics and their incidence in singleton and monozygotic splitting groups

Age and specific cycle characteristics	Singleton (n=845)	MZT (n=14)	*p*-value
**Age** *Mean ± SD* Maternal Donor Paternal	39.93±4.96 24.77±3.74 41.74±7.08	38.42±5.27 23.50±4.95 38.67±4.04	0.295^a^ 0.632^a^ 0.452^a^
**Source of the eggs** n (%) Own Donated	273 (32.3) 572 (67.7)	6 (42.9) 8 (57.1)	0.403^b^
**Type of ET cycle** n (%) Fresh Frozen	455 (53.8) 390 (46.2)	4 (28.6) 10 (71.4)	0.060^b^
**PGT-A** n (%) - +	601 (71.1) 244 (28.9)	5 (35.7) 9 (64.3)	0.004^b^

a Student's test

b Pearson's chi-squared test (χ^2^)

Table 2 shows that the incidence of zygotic splitting following PGT was 3.5% (95% CI 1.8% - 6.6%) *versus* 0.8% (95% CI 0.3% - 1.9%) following ICSI (without embryo biopsy).

**Table 2:** Risk MZT

PGT-A n (%)	MTZ Risk (95% IC)
-	0.825 (0.353 - 1.917)
+	3.557 (1.883 - 6.621)

Table 3 presents a logistic regression analysis evaluating the effect of age and specific cycle parameters over the incidence of MZT. In the univariate analysis, cycles using donated eggs were more associated with a significant increased risk in monozygotic splitting when compared to own-eggs ICSI cycles (OR 5.01, 95% CI 1.1 - 22.69). The transfer of a frozen embryo also resulted in a significantly increased risk for MZT when compared to a fresh embryo in the univariate analysis (OR 4.12, 95% CI 1.04 - 16.18). After adjusting for these potential confounders (source of eggs and fresh/frozen cycles), the PGT procedure was associated with a significant increase in the incidence of MZT when compared to a non-biopsied embryo (OR 3.44, 95% CI 1.05-11.27, *p*=0.041).

**Table 3:** The association between ART parameters and MZT

ART parameters	OR (95% CI)	*p*-value
**Source of the eggs** Own Donated	4.121 (0.436-13.450) 5.011 (1.107-22-694)	0.312^a^ 0.036^a^
**Type of ET cycle** Fresh Frozen	3.417 (0.476-24.560) 4.121 (1.049-16.187)	0.222^a^ 0.043^a^
**Global**	3.448 (1.054-11.279)	0.041^b^

a Univariate logistic regression

b Adjusted multivariate logistic regression. Confounding factors: Source of the eggs and type of ET cycle

Table 4 shows a sub-analysis considering blastocyst-stage transfers only in the control group. The frequency of monozygotic splitting remains statistically significant (*p*=0.006) in PGT cycles as occurred in the overall analysis (Table 1). However, after adjusting for the described potential confounders (source of the eggs and type of ET cycle), the PGT group was more frequently associated with MZT, but without reaching statistical significance (*p*=0.066).

**Table 4:** Incidence in singleton and monozygotic splitting groups only on blastocyst-stage transfers (D+5/D+6)

blastocyst	Singleton	MZT	*p*-value
	(n=775)	(n=14)	
**PGT-A** n (%) - +	536 (69.2) 239 (30.8)	5 (35.7) 9 (64.3)	0.006^a^
**Global** OR (95% CI)	3.113 (0.928-10.443)		0.066^b^

a Pearson's chi-squared test (χ^2^)

b Adjusted multivariate logistic regression. Confounding factors: Source of the eggs and type of ET cycle

## DISCUSSION

To the best of our knowledge, this is the first study focused on exploring the potential effect of the embryo biopsy procedure at the blastocyst stage vis-a-vis the incidence of monozygotic twin gestations. Notwithstanding the weaknesses associated to its retrospective design (including residual confounding) and limited sample, still our findings can contribute to the body of (scarce) medical evidence on the subject. We report an increased risk of monozygotic splitting with the PGT embryo biopsy procedure when performed at blastocyst stage in ART cycles.

The main strength of our study is the inclusion of cycles receiving only a single embryo for transfer and a vast proportion of embryos transferred at the blastocyst stage, thereby limiting the potential bias associated with the transfer of multiple embryos at different embryonic stages.

Even after single embryo transfer, we had a 1.6% monozygotic twin rate, higher than what is reported after spontaneous conception (0.40-0.45%; Aston *et al*., 2008), but similar to previous reports in ART cycles (1.4%, Nakasuji *et al*., 2014); 2.2%, Mateizel *et al*., 2016) including PGT only cycles (1.9%, Verpoest *et al*., 2009). Even though the absolute numbers are quite small, these findings additionally confirm that ART constitutes a risk factor for monozygotic pregnancies.

More specifically, within these SET cycles only, we considered exploring for certain cycle parameters and their possible association with embryo splitting; the source of the eggs: own vs donated (proxy for the potential impact of age), fresh vs frozen embryo transfer cycle and TE biopsy for PGT. According to our data, donor-eggs appear to be associated with an increased risk of MZT. This finding is concordant with a previous publication (Luke *et al*., 2014), but in disagreement with a previous meta-analysis finding no association between the use of donor-eggs and the risk of MZT (Busnelli *et al*., 2019), among the possible explanations for these discrepancies are the number and stage of the embryos transferred from oocyte-donors in other studies which (together with other residual confounders) may introduce a source of bias. Interestingly, in the case of own-eggs cycles, the meta-analysis by Busnelli *et al*. (2019) found that embryos derived from younger oocytes (i.e., female age at time of retrieval <35 years) were significantly more likely to result in an MZT pregnancy. Nonetheless, blastocyst transfer was considered as a potential confounding factor, since it is well known that embryos derived from young oocytes are more likely to be transferred at an advanced blastocyst stage, finally concluding that age may not be an independent risk factor for MZT but rather a proxy for blastocyst transfer (Busnelli *et al*., 2019). However, in our study, the vast majority of embryos (93.3%) were transferred at the blastocyst stage and still donor-eggs remained associated to an increased risk for monozygotic splitting. As an additional note, one previous study has reported that two MZT resulted from the transfer of embryos derived from a single oocyte donor's retrieval and also another oocyte recipient who underwent a two-ET conceived quadruplets (Knopman *et al*., 2014). Further data is warranted to deepen in the exact etiology behind the association of younger age and MZT, beyond the (probwably) valid but unspecific: “healthier oocytes and superior reproductive potential” argument (Knopman *et al*., 2014).

MZT is a low rate event; thus, a high number of cases should be analyzed to find significant differences. In terms of embryo stage upon ET, since the blastocyst stage embryo transfer is highly predominant in our center, we expected a vast majority of the control cases to fit into the blastocyst stage transfer category. Indeed, the vast proportion of transfers in the control group (93.3%) were in the blastocyst stage. To expand on the issue, we performed statistics including blastocyst-stage transfers only day-5/day-6 (n=775), which resulted in similar outcomes in the crude analyses, consistently showing an increase in the MZT rate associated with the embryo biopsy procedure (Table 4). Nonetheless, -and presumably as a consequence of the reduction in sample size-, the significance is lost when accounting for confounders in the data analysis, even though a trend is evident. Taken all together, even acknowledging the absence of statistical significance in the blastocyst-only sub-analysis, our data is still concordant with an increased risk for MZT associated with the embryo biopsy procedure.

Regarding fresh *vs.* frozen cycles, our study found that the transfer of a frozen embryo was associated with an increased risk of MZT in the univariate analysis. The same finding has been reported by several (Alikani *et al*., 2003; Knopman *et al*., 2014; Nakasuji *et al*., 2014; Ikemoto *et al*., 2018) but not all previous publications (Mateizel *et al*., 2016; Busnelli *et al*., 2019) on the topic. It is worth noting that a significant proportion of PGT cycles in our study were transferred in a frozen cycle (148/253, 58.5%). Hence, the possibility exists that the frozen cycle may act as a confounding factor. Consequently, frozen cycles might not be a true independent risk factor for MZT, but rather a proxy for PGT impact. An important limitation of our observational study is that data regarding frozen-warmed embryo transfer cycles did not include information about specific endometrial preparation protocols, which may have influenced the outcome (e.g., an endometrial preparation in a natural cycle may have contributed to an “additional” natural conception). Therefore, a causal/casual relationship between zygotic splitting and embryo-cryopreservation-frozen/thaw ART procedures might be suggested, but cannot be demonstrated from our data.

Embryo PGT entails several invasive procedures and manipulations including breaks, (laser-assisted) intentional holes in the ZP and TE cells biopsy, all of which could potentially increase MZT rates (Verpoest *et al*., 2009). According to our data, even after adjusting for potential confounders, PGT cycles remain a significant factor associated with an increased risk for monozygotic splitting compared to ICSI cycles. Our findings contradict a previously reported data on the topic, which showed no increase in MZT rate and PGT-A compared to regular ICSI after fresh blastocyst transfer (Verpoest *et al*., 2009). This divergence may be attributed to several factors. First, the number of embryos for transfer. In the study by Verpoest *et al*. (2009), a mean of 1.6 embryos (in each arm) were transferred and, -as acknowledged by authors-, chorionicity was not clearly established in MZT pregnancies. This policy may have introduced a substantial bias since a causal relationship between the higher number of embryos transferred and MZT has been argued (Sills *et al*., 2000). In our study, albeit chorionicity or DNA fingerprinting were also not addressed, the inclusion of SET only cycles would have limited this potential source of bias. Secondly, the inclusion of fresh-only cycles by Verpoest *et al*. (2009); as previously discussed, almost 60% of PGT cycles in our study included frozen-thawed cycles, which may have additionally contributed to the MZT outcome as suggested in the univariate analysis. Specific changes in the ZP or TE cells during/after vitrification in a blastocyst-stage biopsied embryo could be hypothesized as potential additional factors associated with the increased risk of MZT in frozen cycles.

This hypothesis warrants further investigations. Third, and of the utmost importance, the difference in the embryo-stage when performing the biopsy. The study by Verpoest *et al*. (2009) included cleavage-stage embryo biopsies, which is a less invasive approach if compared with the TE blastocyst stage embryo biopsy. Additional to the procedures for cleavage-stage biopsies, blastocyst biopsies add aspiration and laser-assisted excision in combination with mechanical removal of TE cells by “pulling” or “flicking”. A possible factor explaining the split of the inner mass cell (IMC) during long culture up to the blastocyst stage for subsequent embryo biopsy is the exposure of the embryo to low concentrations of Ca^+2^ in the culture media. These low concentrations may promote less cells-adhesion and a “weakening” of the inter-cellular junctions provoking the detachment of some cells from the IMC and the formation of an extra embryonic pole in the TE (Milki *et al*., 2003). Therefore, it can be hypothesized that the exposure of the embryo to low Ca^+2^ concentrations together with the mechanical manipulation during embryo biopsy may act as added factors (chemical and mechanical) favoring a later splitting of the ICM, resulting in MZT. While some reports may support this hypothesis (Edwards *et al*., 1986; Van Langendonckt *et al*., 2000; Yan *et al*., 2015), a recent retrospective evaluation focused on PGT cycles (Gu *et al*., 2018) taking into account the “hatching” status of the blastocyst (partially and fully hatched blastocysts vs 8-shaped blastocysts with ICM incarceration) before biopsy and the relation with embryo splitting, showed that ICM incarceration in 8-shaped blastocysts did not increase MZT incidence. While some differences in the protocol may explain this discrepancy, Gu *et al*. (2018) still reported an overall MZT pregnancy rate per established clinical pregnancy of 2.8% (similar to our rate). Unfortunately, the study did not include regular ICSI with blastocyst transfer for comparison. In concordance with our study, the (additional) influence of AH in the MZT rate could not be disentangled in the study by Gu *et al*. (2018), because all the embryos in the PGT group received AH on day 3 of embryo development. But, as suggested by some observations, even if the zona manipulation on cleavage-stage (i.e., AH) may be involved in some cases of MZT, it is unlikely to be an exclusive mechanism (Gu *et al*., 2018). Nonetheless, in the study by Verpoest *et al*. (2009) cleavage stage embryo-biopsies did not show correlation with an increase in MZT rate; in these regards, cleavage-stage biopsy could be inferred as a proxy for AH (both procedures involves “making a hole” in the zona pellucida during cleavage stage).

Also, some additional drawbacks from our study merits further analysis. MZT is - fortunately- an infrequent event; indeed studies focused on rare phenomenon constitute a challenge. Obviously, the results from our study, with a limited sample size, should be further explored in large databases in order to reach robust conclusions. However, due to the sharp increase in PGT cycles worldwide, even small increases in incidences may translate into important crude numbers of this potentially complex obstetric condition. The results from our study may inspire future studies on the subject. The lack of confirmation of monozygocity at birth constitutes another arguable limitation of the study; we believe that this approach is only of marginal importance, and it is not crucial for the purpose of diagnosis of am MZT (especially after SET policy); several publications on the topic have clearly established that transvaginal ultrasound is highly accurate in diagnosing a MZT pregnancy; moreover all cases in our study received one additional and dedicated evaluation of the MZT status on the week after the initial visualization for confirmation (weeks 6-7). Notwithstanding these potential limitations, still the results found in our study can contribute to the body of medical evidence on the subject.

## CONCLUSION

Our findings indicate that embryo biopsy for PGT performed at the blastocyst stage is associated to an increase in the incidence of MZT. Because of the increased risk associated with MZT, this information could be useful to help identify potential factors underlying the higher incidence of MZT in the context of ART, and to identify strategies to reduce this incidence. Given the low event rates with monozygotic twinning, our results warrant validation in larger sample size studies required to provide higher statistical power. Until those studies become available, patients undergoing PGT must receive proper counselling about the potential risks of the technique.
